# From E. G. B.

**Published:** 1882-05

**Authors:** 


CINCINNATI, April 1, 1882.

EDITOR REGISTER:—For the past few years it has been apparent to many members of the profession that our dear old mother, the Mississippi Valley Dental Association, is dying of general debility. There are many reasons for this, no doubt; principally the large number of State and local societies that have sprung into existence since the old lady taught us *how* to conduct a society. Very many of the men engaged in practice at the present day learned their elementaries at her annual meetings, and with shameful ingratitude are now giving the results of their training and experience to swell the importance of pretentious local bodies that are as yet scarce dry behind the ears. The result has been to deplete the M. V. D. A. of nearly all the vigorous blood that once nourished the new-born science, and made her heart-throbs audible to all in the land.

Not least potent in bringing about this disgraceful state of things, is the assinine persistence of some unmentionables to invariably interrupt the order of business, break up the discussion at an interesting stage, merely to foist upon the attention of the members, either their own impotent twaddle or that of some windy edition of antiquity.

The past meeting of the Association promised better than those of some years back. The young men had taken the bit between their teeth and with much difficulty had secured an essayest to open each subject with a paper. The programme was arranged with intelligence, and was the only one for some time past that displayed anything like continuity of idea in the sequence of topics.

But no; this legitimate and worthy effort to revivify, was too much for the ponderous brains of the insatiable kicker. The discussion of an important, interesting paper was squelched, another paper rushed in ahead of time, all to prepare for a cyclone from Chicago, that should have had at least *some* relevancy to the subject in hand. But this is not all; the society was cheated out of another paper that was, at all events, in part prepared.

If the level-headed members of the old association have its interest at heart, they will see to it that every paper costing time and hard work to compose, is given its proper place in the programme. The discussions must *not* be interrupted nor their order changed at the instance of anyone, if we expect to render our meetings profitable, and encourage the writers (for they are few) to give us their best efforts. E. G. B.

